# Seasonal and spatial trends of rickettsioses in Sri Lanka (2009–2023): Spatio-temporal analysis and climate-linked modeling

**DOI:** 10.1371/journal.pone.0357006

**Published:** 2026-08-27

**Authors:** Nayana Gunathilaka, Deshaka Jayakody, Iranga Dilshan

**Affiliations:** Department of Parasitology, Faculty of Medicine, University of Kelaniya, Ragama, Sri Lanka; Christian Medical College Vellore, INDIA

## Abstract

**Background:**

Rickettsioses are caused by obligate intracellular bacteria of the order Rickettsiales, which includes several genera such as *Rickettsia*, *Orientia*, *Anaplasma*, *Ehrlichia*, *Neoehrlichia*, and *Neorickettsia*, and are significant yet under-recognised vector-borne febrile illnesses in Sri Lanka. Despite being a notifiable disease, gaps in understanding its spatial and temporal distribution hinder effective control and prevention. Therefore, the present study aimed at determining long-term epidemiological trends and environmental determinants of rickettsioses incidence in Sri Lanka between 2009 and 2023.

**Methods:**

A retrospective ecological analysis was conducted using weekly rickettsioses case reports obtained from the Epidemiology Unit of the Ministry of Health, Sri Lanka, covering all 25 districts over the period 2009–2023. Mid-year district population data and district land area were retrieved from the Department of Census and Statistics to calculate incidence rates per 100,000 population. Meteorological variables (mean, maximum, and minimum temperatures; total rainfall; and mean relative humidity) were retrieved using NASA POWER satellite-derived datasets. A Zero-Adjusted Gamma (ZAGA) model within the Generalized Additive Model for Location, Scale, and Shape (GAMLSS) framework was utilized to estimate the influence of climatic and spatial variables on incidence rates, with environmental predictors selected based on a priori biological hypotheses regarding vector ecology.

**Results:**

Between 2009 and 2023, a total of 18,486 rickettsioses cases were reported. The highest case burden was detected in Jaffna (n = 7,080), followed by Kandy (n = 1,363), Badulla (n = 1,319), Nuwara Eliya (n = 1,141), Hambantota (n = 1,066), and Monaragala (n = 1,008). The higher incidence rates were concentrated in the Northern and Central highland districts compared to low-incidence regions in the Western and Southern provinces. Temporal analysis indicated seasonal peaks coincide with monsoonal rainfall, with a rise of cases during January to February and September to November. The GAMLSS model demonstrated that mean temperature, relative humidity, and rainfall significantly influenced incidence rates (*P* < 0.05), with high humidity and post-monsoonal rainfall correlating with increased risk. Spatial predictors (latitude and longitude) also contributed to explaining distribution patterns, highlighting localized ecological drivers.

**Conclusions:**

This study provides the first comprehensive nationwide analysis of rickettsioses incidence in Sri Lanka, highlighting firm spatial heterogeneity and clear climatic associations. The findings emphasize the necessity of targeted surveillance and early diagnostic capacity in high-burden districts, particularly Jaffna and the central highlands. Integrating climatic data into surveillance systems may improve early warning and outbreak preparedness. Improved diagnostic availability at regional hospitals and enhanced physician awareness are essential to reduce under-diagnosis and guide timely interventions.

## Background

Rickettsioses is one of the leading causes of vector-borne febrile illness in Asia, transmitted through arthropod vectors such as ticks, mites, fleas, and lice [1,2]. Rickettsioses are caused by obligate intracellular bacteria of the order Rickettsiales, which includes several genera such as *Rickettsia*, *Orientia*, *Anaplasma*, *Ehrlichia*, *Neoehrlichia*, and *Neorickettsia* [3,4]. Several distinct forms of rickettsioses have been described: the spotted fever group (SFG), the typhus group (TG), and scrub typhus [5]. Globally, the SFG comprises over 30 species, including *R. rickettsii* (Rocky Mountain spotted fever, North America), *R. australis* and *R. honei* (Australia), and *R. conorii* (Mediterranean spotted fever, Europe, Africa, and Asia) [6–9]. The TG includes *R. typhi* (murine typhus) and *R. prowazekii* (epidemic louse-borne typhus) [10], while scrub typhus is caused by *Orientia tsutsugamushi* [11].

In Sri Lanka, rickettsial infections vary regionally. Most cases of SFG rickettsiosis are reported from the western slopes of the central hills, while scrub typhus predominates in the Western, Northern, North Central, and North Western provinces [12–15]. The Southern Province of Sri Lanka exhibits a combination of scrub typhus and spotted fever [16]. Historically, the first records of rickettsial disease in Sri Lanka date back to World War II, when cases of scrub typhus were detected in the Eastern Province [17,18]. Murine typhus was reported as early as 1938 [19].

Currently, rickettsioses are nationally notifiable diseases, with an average of 1,500 cases reported annually in the Weekly Epidemiological Reports of the Ministry of Health [20]. However, accurate clinical diagnosis is challenging, since rickettsial infections share overlapping features with other febrile illnesses common in the tropics, and eschar—a key diagnostic clue is often absent [20]. Clinical presentations range from mild fever to severe, life-threatening illness with significant morbidity and mortality [21–23,]. Early treatment with doxycycline can dramatically reduce complications and deaths [24–28], but diagnostic facilities remain limited in Sri Lanka. Currently, testing is largely confined to a few research laboratories supported by investigator-driven grants and international collaborations [13].

Environmental factors are known to modulate rickettsial disease transmission through well-defined biological mechanisms, providing clear a priori hypotheses for this study. Temperature influences the activity, survival, and developmental rates of arthropod vectors across rickettsial disease types. Trombiculid mite (chigger) larvae the primary vectors of scrub typhus show peak activity during cooler transitional seasons rather than at temperature extremes, since high temperatures accelerate desiccation and mortality [29,30]. Consistent with this, scrub typhus outbreaks in South and East Asia have been repeatedly associated with cooler seasonal windows [31–33]. We therefore hypothesized that mean temperature would be negatively associated with incidence, while temperature extremes (range) would capture transitional seasonal effects positively. Rainfall promotes dense scrub vegetation and moist leaf litter optimal microhabitats for trombiculid mite populations while simultaneously supporting rodent reservoir populations through increased food availability [25,34]. We therefore hypothesized a positive association between rainfall and incidence. Similarly, relative humidity supports mite larval survival by preventing desiccation, and we hypothesized a positive humidity-incidence relationship. For murine typhus, rat flea (*Xenopsylla cheopis*) activity is modulated by temperature and humidity, with survival reduced at temperature extremes, providing an additional mechanistic basis for climate-disease associations [33,35].

Given these complexities, understanding spatio-temporal patterns of rickettsioses in Sri Lanka is essential for disease control. Analyzing long-term case trends can help identify hotspots, evaluate interventions, and predict outbreaks in relation to environmental and climatic variables. Therefore, this study was aimed to analyze the spatio-temporal distribution of rickettsioses in Sri Lanka from 2009–2023 using advanced statistical modeling. Therefore, the present study was conducted to identify epidemiological trends and explore associations with climatic drivers to provide insights in supporting early warning systems and targeted interventions.

## Method

### Study setting

Sri Lanka is a tropical island in the Indian Ocean, covering approximately 65,610 km² and administratively divided into 26 districts. Sri Lanka marked geographical diversity, featuring flat coastal plains and mountainous central highlands that exceed 2,524 meters above sea level. Climatic conditions in the country influence mainly by two annual monsoon seasons: the southwest monsoon (May to September) and the northeast monsoon (December to February). The wet zone receives ~2,500 mm of annual rainfall, while the dry zone receives ~1,200 mm. Temperatures range from 17 °C in the highlands to 33 °C in coastal regions.

### Data sources and collection

Weekly rickettsioses case records (January 2009 to December 2023) were obtained for all 26 districts from archived Weekly Epidemiology Reports of the Epidemiology Unit, Ministry of Health, Sri Lanka. Mid-year population estimates by district were retrieved from the Department of Census and Statistics of Sri Lanka. Meteorological data; mean, maximum, and minimum temperatures; total rainfall; and mean relative humidity were obtained monthly using the nasapower package in R (version 4.4.0), which accesses NASA’s POWER (Prediction of Worldwide Energy Resources) satellite database (Sparks, 2018).

### Diagnostic basis and disease categorization

In Sri Lanka’s national surveillance system, rickettsioses are notified under the consolidated category of “typhus fever” by the Epidemiology Unit, Ministry of Health, without etiological differentiation between scrub typhus (*Orientia tsutsugamushi*), murine typhus (*Rickettsia typhi*), or spotted fever group (SFG) Rickettsia species. Case notification is based primarily on clinical-epidemiological criteria: acute febrile illness with compatible exposure history (rural, scrubland, or peri-domestic environments), eschar when present, and/or clinical suspicion of rickettsial disease in the absence of confirmed alternative diagnoses. Laboratory confirmation using indirect immunofluorescence assay (IFA), Weil-Felix agglutination, or PCR is available only at a small number of research laboratories and is not routinely incorporated into the national surveillance system.

### Incidence rate calculation

The incidence rate of rickettsioses was calculated for each district and year using the formula:


$$\begin{array}{c} \text{Incidence rate}=\frac{\text{Total Number of new cases}}{\text{Total number at risk}}\times \text{100 000} \end{array}$$

In here, the total number of new cases represented the district-level reported cases of rickettsioses during the study period, while the mid-year population was used as the denominator to represent the population at risk. The incidence rate was expressed per 100,000 population to allow for comparison across districts and time periods.

### Modeling of rickettsioses case incidence

Rickettsioses incidence rates were analyzed using a Zero-Adjusted Gamma (ZAGA) distribution within the Generalized Additive Model for Location, Scale, and Shape (GAMLSS) framework. This modelling approach was chosen to effectively address the substantial zero inflation in the rickettsioses incidence data, as 38.87% of reported cases had zero incidence rates. In this model, the incidence rate was treated as the response variable. Environmental parameters (mean relative humidity, mean temperature, maximum temperature, minimum temperature, and total rainfall) were included as predictor variables, each selected on the basis of a priori biological hypotheses regarding their role in modulating rickettsial vector ecology, as described in the background. To capture spatial effects, the latitude and longitude of the district centers were used, while year and month were incorporated to account for temporal variation. The ZAGA spatial GAMLSS model consists of three linked equations that model: the expected incidence rate (μ), the dispersion parameter (α), and the zero-inflation probability (ν), each as smooth functions of the predictor variables.

(1) Expected Incidence Rate Model

$$\begin{array}{cc} \text{log}\left(\mu_{i}\right)= & \beta_{\text{0}}+f_{\text{1}}({meanrelativehumidity}_{i})+f_{\text{2}}(meant{emperature}_{i})+f_{\text{3}}({maximumtemperature}_{i})\\ & +f_{\text{4}}(m{inimumtemperature}_{i})+f_{\text{5}}({totalrainfall}_{i})+f_{\text{6}}(y{ear}_{i})+f_{\text{7}}(m{onth}_{i})\\ & +f_{\text{8}}(l{atitude}_{i})+f_{\text{9}}({logitude}_{i}) \end{array}$$

(2) Dispersion Model

$$\begin{array}{cc} \text{log}\left(\alpha_{i}\right)= & \gamma_{\text{0}}+f_{\text{1}}({meanrelativehumidity}_{i})+f_{\text{2}}(meant{emperature}_{i})+f_{\text{3}}({maximumtemperature}_{i})\\ & +f_{\text{4}}({mimimumtemperature}_{i})+f_{\text{5}}({totalrainfall}_{i}) \end{array}$$

(3) Zero-Inflation Model

$$\begin{array}{c} \text{log}\left(\frac{v_{i}}{\text{1}-v_{i}}\right)=\theta_{\text{0}}+g_{\text{1}}({latitude}_{i})+g_{\text{2}}({longitude}_{i}) \end{array}$$

For district “i”,

$\text{log}\left(\mu_{i}\right)$ = log-transformed expected incidence rate

$\text{log}\left(\alpha_{i}\right)$ = log-transformed dispersion parameter of the Gamma distribution

$\text{log}\left(\frac{v_{i}}{\text{1}-v_{i}}\right)$= log-odds of zero incidence due to zero-inflation.

$\beta_{\text{0}},\gamma_{\text{0}}$ ${and\theta }_{\text{0}}$are intercept terms. $f_{\text{1}}$through $f_{\text{9}}$ and $g_{\text{1}}$, $g_{\text{2}}$ represent smooth functions.

The goodness-of-fit of the model was assessed by examining the distribution of the residuals. All data were analyzed using R 4.4.2 software, employing the gamlss, mgcv, sp, ggplot2, dplyr, readr gamlss.dist and forcats packages.

### Ethics statement

Ethical approval was not required for this study because the analysis was conducted exclusively using publicly available, secondary data obtained from official sources. No human participants were directly involved and no identifiable personal information was collected or analyzed.

## Results

### Rickettsioses case incidence

**Fig 1:**
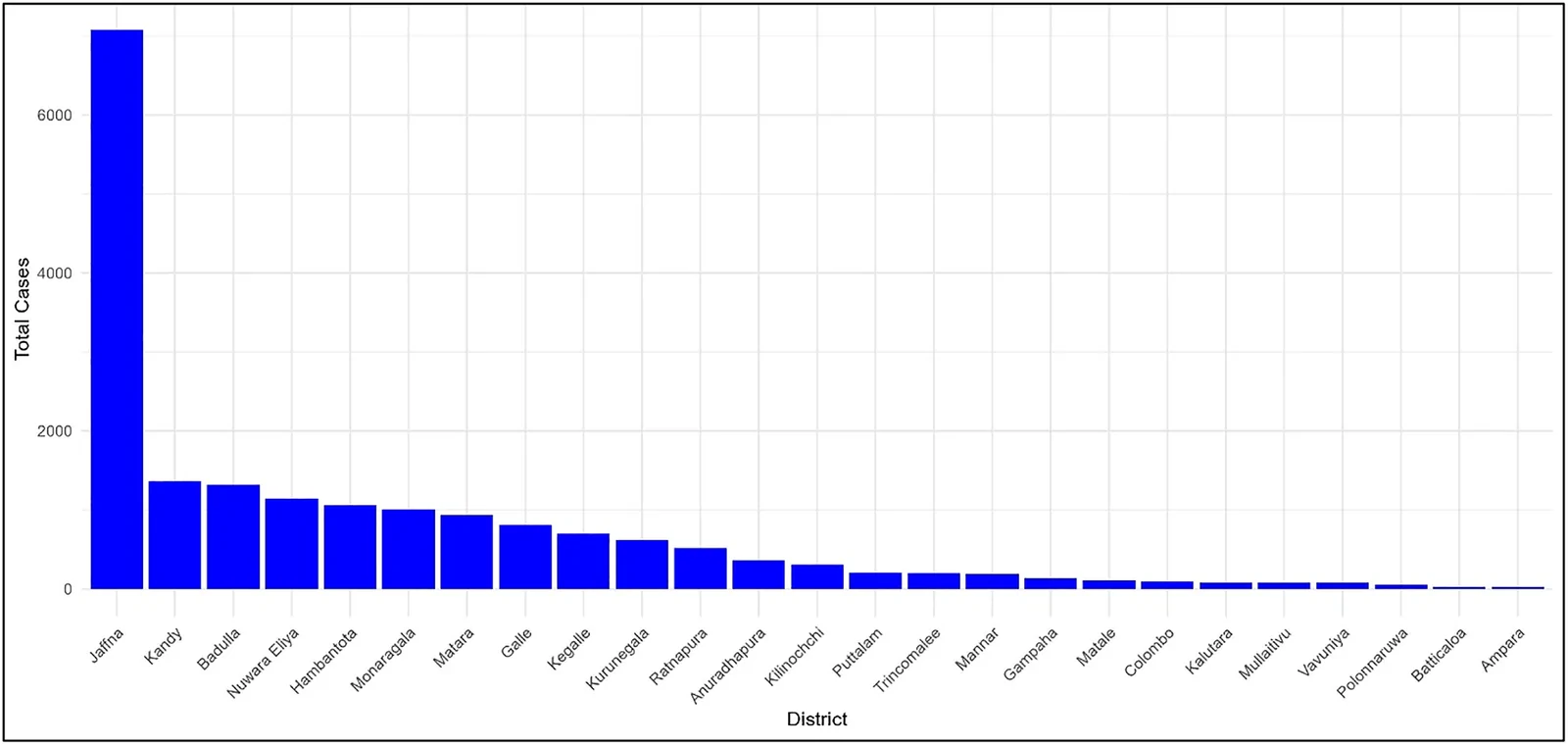
Rickettsioses cases reported in Sri Lanka from 2009 to 2023.

During the study period, a total of 18,486 rickettsial cases have been reported in Sri Lanka (Fig 1). The distribution of cases was highly heterogeneous across districts, with certain areas contributing disproportionately to the national case burden. The Jaffna District in the northern province of Sri Lanka reported the largest number of cases (n = 7,080; 38.3% of total cases), indicating a substantial endemic focus in the Northern Province. Other districts with high case counts included Kandy (n = 1,363; 7.4%), Badulla (n = 1,319; 7.1%), Nuwara Eliya (n = 1,141; 6.2%), Hambantota (n = 1,066; 5.8%), and Monaragala (n = 1,008; 5.5%). Together, these six districts accounted for more than two-thirds of the national caseload during the study period.

### Spatial heterogeneity of rickettsioses cases in Sri Lanka

**Fig 2:**
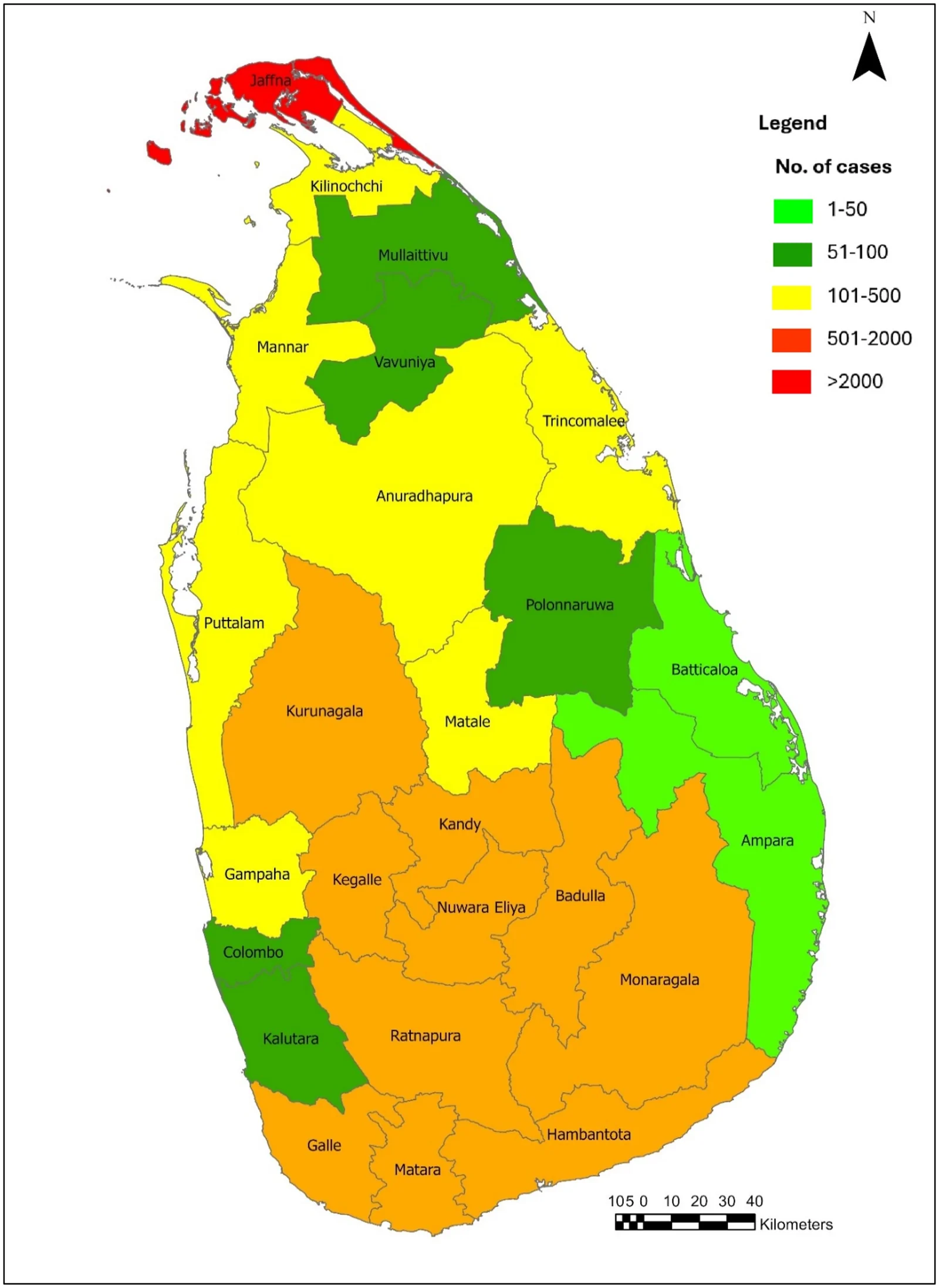
Distribution of rickettsioses cases across administrative districts in Sri Lanka, 2009–2023. Map generated in ArcGIS Pro using the Humanitarian Data Exchange basemap of Sri Lanka administrative boundaries (https://data.humdata.org/dataset/sri-lanka-administrative-levels-0-4-boundaries).

There was a marked spatial heterogeneity of the distribution of rickettsioses cases across districts, with geographic variation in disease burden (Fig 2). The Northern Province, particularly the Jaffna District, emerged as the single largest focus of rickettsioses in the country. The central highlands including Kandy (n = 1,363), Badulla (n = 1,319), and Nuwara Eliya (n = 1,141) formed another cluster of high incidence suggesting localized ecological or socioeconomic risk factors. The districts in the Southern province of Sri Lanka, especially Hambantota (n = 1,066) and Monaragala (n = 1,008), also reported a substantial number of cases. In contrast, many districts in the Western and urbanized regions recorded comparatively fewer cases.

### Key predictor effects in ZAGA GAMLSS model

The mean temperature had a significant negative association with incidence rates (estimate = –0.695, *P* < 0.05), indicating that higher mean temperatures associated with lower rickettsioses incidence rates (Table 1). In contrast, both maximum temperature (estimate = 0.334, *P* < 0.05) and minimum temperature (estimate = 0.215, *P* < 0.05) showed significant positive associations. Total rainfall was also significantly positively associated with incidence rates (estimate = 0.0003, *P* < 0.05). In addition, both latitude (estimate = 0.8453, *P* < 0.05) and longitude (estimate = 0.6504, *P* < 0.05) were significantly positively associated with incidence rates, indicating a geographic pattern in disease distribution.

**Table 1:** Parameter and risk estimates of the expected incidence rate model.

Variable	Estimate	Std. Error	Risk Estimate	95% confidence interval of risk estimate	t value	P value
(Intercept)	−20.69	0.79	–	–	−25.952	0.00
pb(mean relative Humidity)	0.00	0.00	1.00	0.99 −1.01	1.12	0.26
pb(mean temperature)	−0.69	0.03	0.49	0.46 - 0.53	−18.95	0.00
pb(maximum temperature)	0.33	0.01	1.39	1.34 −1.44	19.09	0.00
pb(minimum temperature)	0.21	0.02	1.23	1.18 - 1.29	9.81	0.00
pb(total rainfall)	0.00	0.00	1.00	–	2.67	0.00
pb(year)	−0.01	0.00	0.98	0.98 - 0.98	−51.31	0.00
pb(month)	0.00	0.00	1.00	0.99 −1.01	0.78	0.43
pb(latitude)	0.84	0.01	2.32	2.27 - 2.38	65.77	0.00
pb(longitude)	0.65	0.00	1.91	1.88 −1.94	90.24	0.00

The relative humidity (estimate = –0.0134, *P* < 0.05) and maximum temperature (estimate = –0.0682, *P* < 0.05) were both significantly negatively associated with variability when examining the variability of incidence rates across districts (Table 2). Other environmental variables, including mean temperature, minimum temperature, and total rainfall, did not have significant effects on variability. The probability of excess zeros in the incidence data, both latitude (estimate = 0.6038, *P* < 0.05) and longitude (estimate = 0.4037, *P* < 0.05) were significantly and positively associated with zero inflation (Table 3).

**Table 2:** Parameter and risk estimates of the dispersion model.

Variable	Estimate	Std. Error	Dispersion ratio	95% confidence interval of risk estimate	t value	P value
(Intercept)	1.77	0.67	–	–	2.62	0.00
pb(mean relative humidity)	−0.01	0.00	0.98	0.97 -, 0.99	−3.06	0.00
pb(mean temperature)	0.01	0.07	1.01	0.87 −1.18	0.23	0.81
pb(maximum temperature)	−0.06	0.03	0.93	0.87 - 0.99	−2.12	0.03
pb(minimum temperature)	0.01	0.04	1.01	0.93 −1.11	0.37	0.70
pb(total rainfall)	−0.00	0.00	0.99	0.99 −1.00	−0.66	0.50

**Table 3:** Parameter and risk estimates of the zero-inflation model.

Variable	Estimate	Std. Error	Odds Ratios	95% confidence interval of risk estimate	t value	P value
(Intercept)	−37.75	7.95	–	–	−4.74	0.00
pb(latitude)	0.60	0.03	1.82	1.69 - 1.97	15.84	0.00
pb(longitude)	0.40	0.09	1.49	1.23 - 1.81	4.07	0.00

### Goodness-of-fit of the model

**Fig 3:**
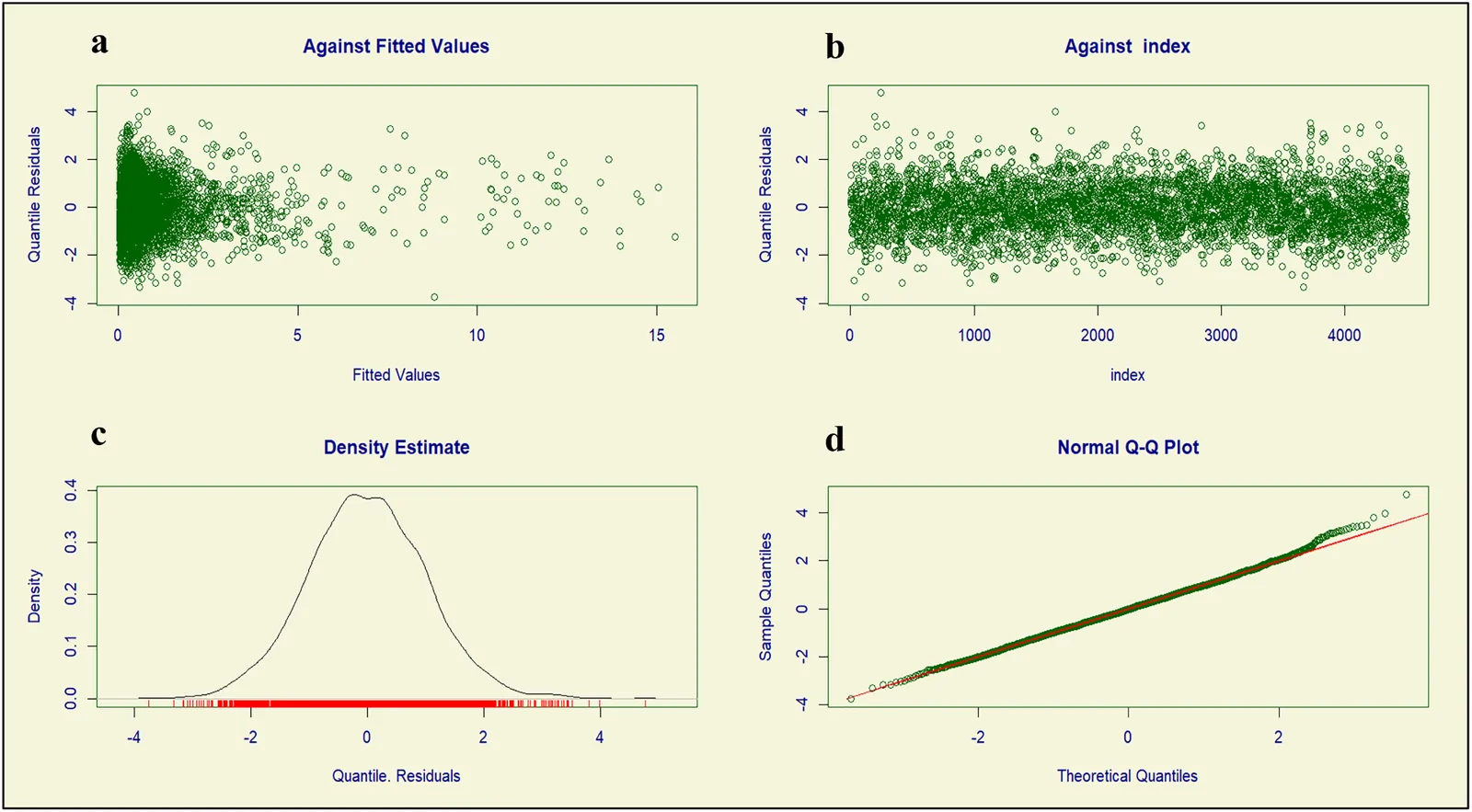
Residual diagnostic plots for evaluating the adequacy of the Zero-Adjusted Gamma (ZAGA) Generalized Additive Model for Location, Scale, and Shape (GAMLSS): (a) Residuals vs. fitted values;(b) Residuals vs. observation index;(c) Density plot of residuals; and (d) Normal Q-Q plot.

According to the Residual diagnostic plots, the Zero-Adjusted Gamma (ZAGA) GAMLSS model provides a good fit to the data. The residuals are randomly scattered around zero when plotted against fitted values (Fig 3a), suggesting no major issues with non-linearity or heteroscedasticity. No visible patterns were observed in the residual plotted against the observation index, indicating the absence of significant autocorrelation (Fig 3b). According to the residuals, the density plot approximates a normal distribution, forming a bell-shaped curve (Fig 3c). The Normal Q-Q plot in Fig 3d confirms this observation, as the residuals closely follow the 45-degree reference line, further supporting the assumption of normality. Taken together, these diagnostic plots do not indicate any critical violations of model assumptions, suggesting that the ZAGA GAMLSS model adequately captures the underlying structure of the data.

### Temporal distribution of rickettsioses incidence

**Fig 4:**
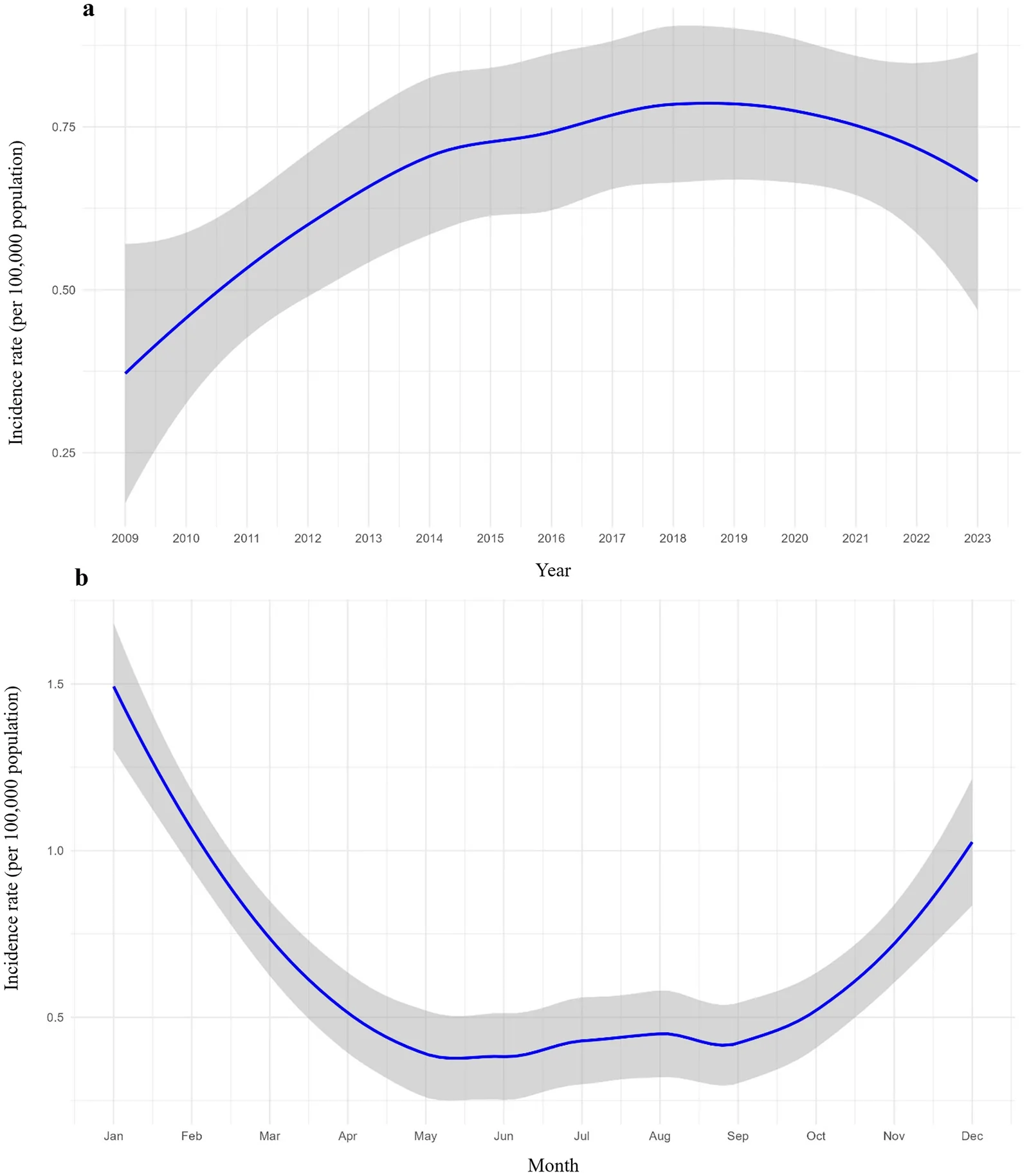
Trend-lines of rickettsioses incidence rates per 100,000 population in Sri Lanka, with shaded areas representing the 95% confidence intervals: (a) annual trends and (b) monthly trends.

According to the annual trend of rickettsioses incidence rate per 100,000 population, the incidence rate showed a steady, gradual increase up to 2019, followed by a decline through 2023 (Fig 4a). The monthly trend indicates that incidence rates were typically highest at the beginning and end of each year (Fig 4b). From January to April, there was a marked decrease, after which the incidence rate remains relatively low until September, followed by a rapid increase towards December (Fig 4c).

### Spatial distribution of rickettsioses disease incidence

**Fig 5:**
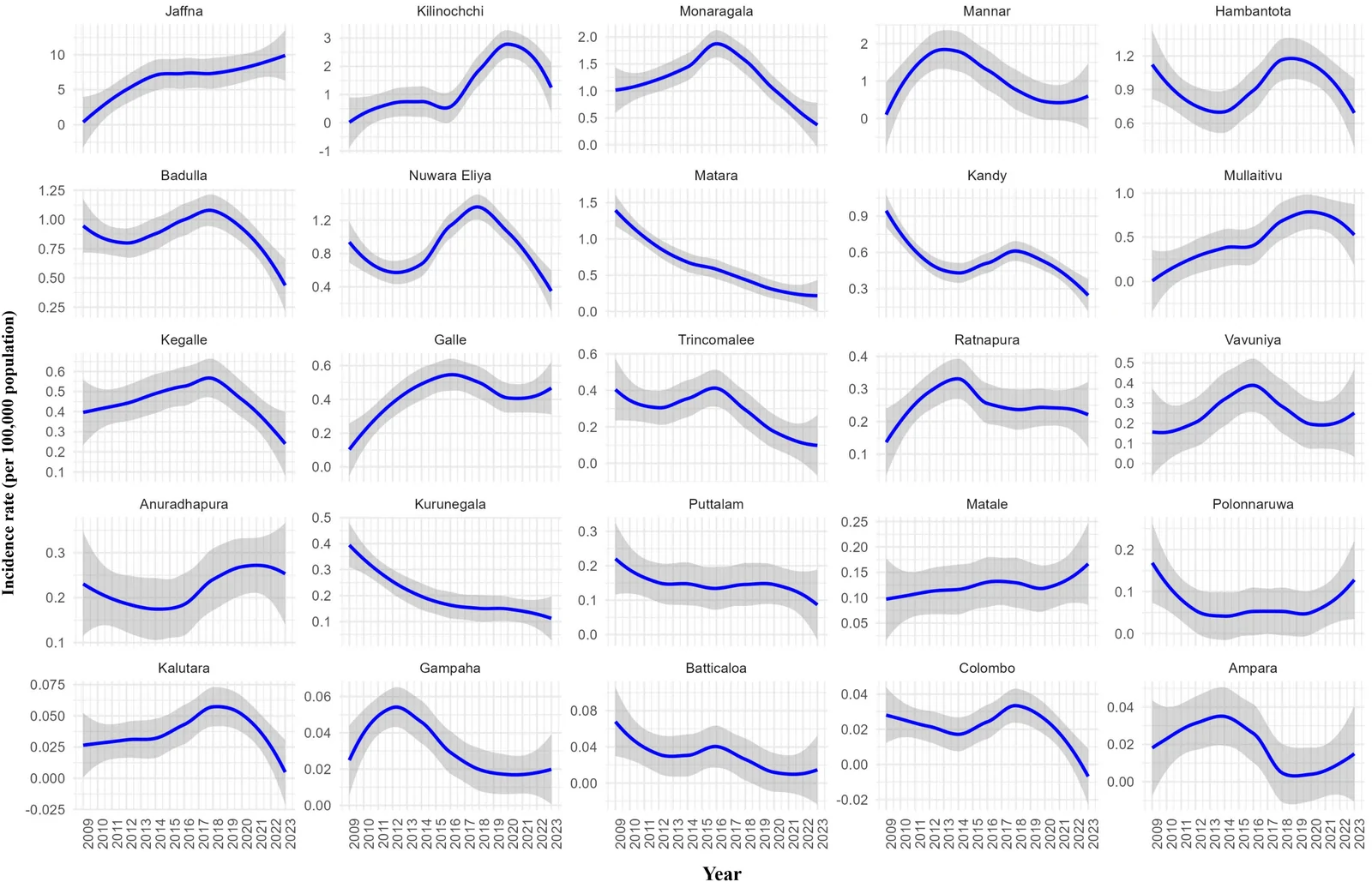
Annual trends in rickettsioses incidence rates (per 100,000 population) by district, Sri Lanka, 2009–2023, with shaded areas representing 95% confidence intervals.

The yearly trend of rickettsioses incidence in Sri Lanka per 100,00 population in each district is illustrated in Fig 5. The Polonnaruwa district experienced an increase in incidence after reaching its lowest levels during 2011–2012, maintaining that low level until 2019–2020; more recently, the incidence has been rising again (Fig 5). A marked decline in the incidence was experienced in Kilinochchi, Hambanthota, Badulla, Nuwara Eliya, Kegalle, Kalutara, and Colombo districts after the peak during 2018–2019 (Fig 5). Monaragala, Trincomalee, and Vavuniya districts showed a decline after peaking around 2015–2016. A gradual decline after peaking around 2012–2013 was observed in Mannar, Ratnapura, Gampaha, and Ampara districts. Matara, Kandy, Kurunegala, Puttalam, and Batticaloa showed an overall decreasing trend with only minor fluctuations (Fig 5).

**Fig 6:**
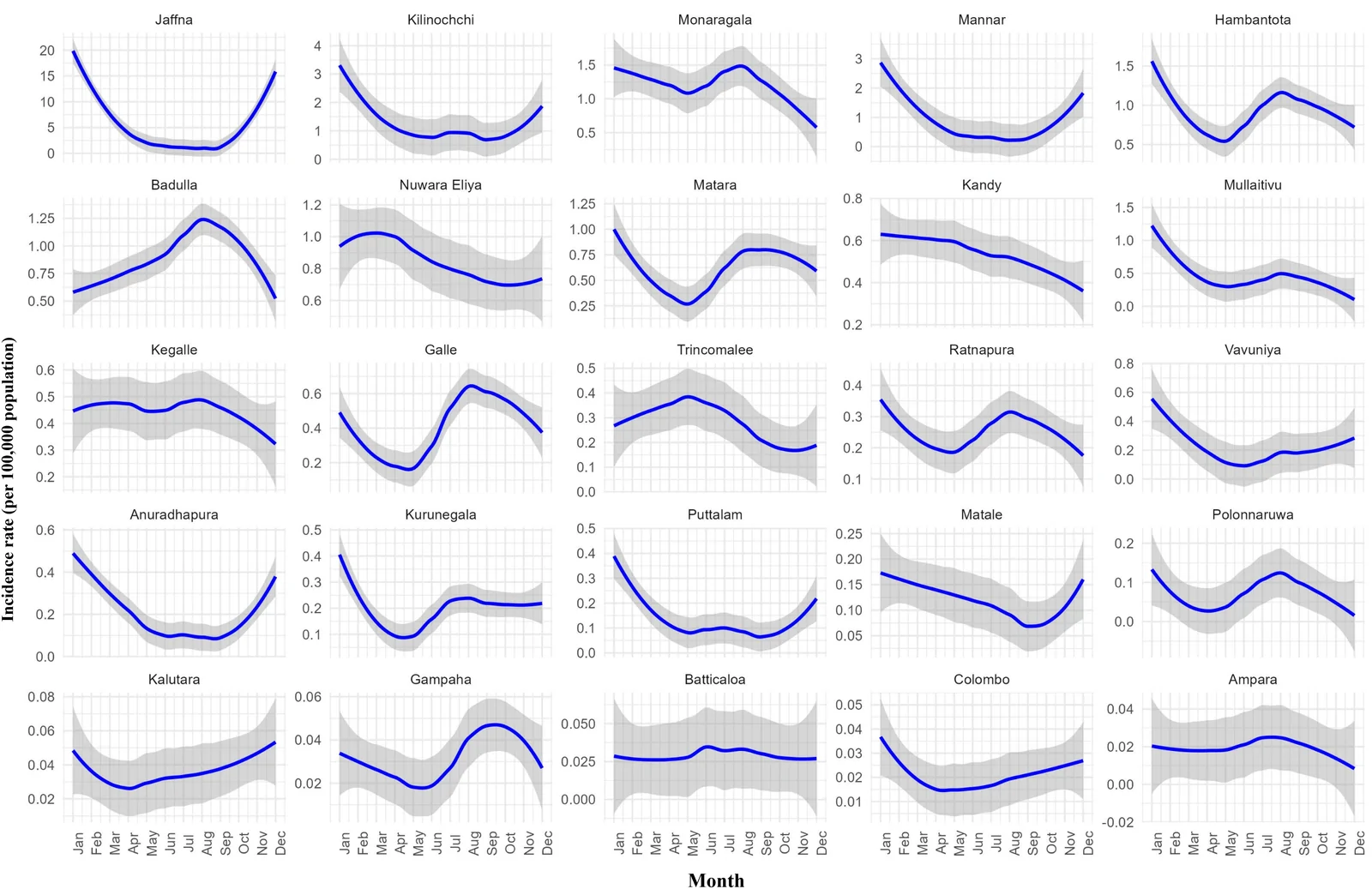
Monthly trends in rickettsioses incidence rates (per 100,000 population) by district, Sri Lanka, 2009–2023, with shaded areas denoting 95% confidence intervals.

The monthly trends in rickettsioses incidence per 100,000 population across districts are illustrated in Fig 6. Jaffna, Kilinochchi, Mannar, Anuradhapura, and Puttalam showed a similar seasonal pattern, with the highest incidence occurring at the beginning and end of the year, and a pronounced decrease during the middle months. The districts of Monaragala, Hambantota, Matara, Galle, Ratnapura, Polonnaruwa, and Gampaha exhibited a decrease in incidence until April, followed by a sharp increase that peaked in August–September, and then a decline towards the end of the year.

In the Badulla district, the peak incidence also occurred in August; however, unlike the previous group, the incidence increased gradually until the peak, followed by a rapid decline. Nuwara Eliya, Kandy, Mullaitivu, Kegalle, and Trincomalee districts showed a gradual decline over the year with only minor fluctuations. In Batticaloa, incidence remained relatively stable throughout the year. Kalutara was the only district showing a steady increase in incidence as the year progressed. Matale district showed a rapid increase after August, following a gradual decline earlier in the year. Kurunegala district recorded peak incidence at the beginning of the year, followed by a sharp decline in March–April, then an increase in June–July that remained steady thereafter.

## Discussion

The present study documents the first comprehensive spatio-temporal and climate-linked analysis of typhus fever in Sri Lanka for a considerable period (over 15-year period from 2009–2023. The findings emphasize a considerable spatial heterogeneity in disease burden, temporal fluctuations in incidence, and distinct associations with climatic variables. Together, these insights emphasize the complex interplay between environmental drivers, geography, and epidemiology of rickettsioses, with important implications for disease surveillance and control. Furthermore, this investigation revealed substantial spatial heterogeneity, seasonal variation, and strong associations with climatic variables, reflecting complex interactions among the environment, vector ecology, and human exposure.

The distribution of cases was highly uneven across districts. The geographic distribution of cases highlights Jaffna as the major endemic focus, contributing nearly 40% of the national caseload. The disproportionate concentration of cases in the Jaffna District is biologically plausible when interpreted in light of the documented ecology of the Northern Province. Jaffna lies within Sri Lanka’s dry zone, where seasonally moist scrub vegetation, intensive smallholder home-garden and field cultivation and widespread goat and cattle rearing generate abundant peri-domestic microhabitats for chigger mite larvae and for the rodent reservoirs that sustain them. The re-establishment and strengthening of agricultural activity on previously under-utilised land in the Northern Province during the post-conflict period may have increased human contact with mite-infested scrubland. In addition, socioeconomic vulnerabilities, including limited access to confirmatory diagnostics and comparatively heightened regional clinical awareness of scrub typhus, may simultaneously sustain transmission and influence the recorded incidence compared with other provinces.

Since the national surveillance data aggregate all rickettsial diseases without etiological differentiation, this spatial pattern must be interpreted in the context of the known geographic partitioning of rickettsial diseases in Sri Lanka. Published serological and clinical evidence consistently documents that scrub typhus (*Orientia tsutsugamushi*, transmitted by trombiculid mite larvae) predominates in the Northern, North Central, and North Western provinces precisely the regions showing the highest incidence in the present study [12–14]. The central highlands cluster (Kandy, Badulla, Nuwara Eliya) is more consistent with spotted fever group (SFG) typus, which have been well-documented in these areas [15,36]. Coastal and peri-urban areas, where murine typhus (*Rickettsia typhi*, transmitted by rat fleas) is more likely to circulate, showed comparatively lower incidence consistent with the generally lower clinical recognition of murine typhus in Sri Lanka’s surveillance system. Thus, while etiological confirmation is unavailable, the observed spatial patterns align well with the independently documented geographic ecology of these diseases, lending ecological coherence to our spatial model results. This localized clustering also mirrors patterns seen in other Asian countries where scrub typhus and spotted fever typhus occur in distinct ecological “hotspots,” linked to rodent–vector interactions, vegetation cover, and land use [25,32,34,37]. The occurrence of high incidence in the Northern Province may reflect ecological conditions favorable for trombiculid mite vector populations, such as the abundance of rodents and chigger mites in peri-domestic environments. In addition, socioeconomic vulnerabilities, including limited access to healthcare and diagnostic facilities, may contribute to under-recognition and late treatment, thereby sustaining transmission.

The central highlands (Kandy, Badulla, Nuwara Eliya) and parts of the Southern Province (Hambantota, Monaragala) also emerged as secondary foci of high endemicity, supporting earlier clinical and seroepidemiological studies that demonstrated a heterogeneous distribution of both spotted fever group and scrub typhus across Sri Lanka [15,36,38]. The districts in the central highlands that characterized with unique climatic conditions such as lower temperatures, heavy rainfall, and dense vegetation, which may support the survival of arthropod vectors such as mites and fleas. Additionally, the agricultural practices and close human–animal interactions common in these areas may increase the exposure risk. Further, cases reported in the Southern province with substantial numbers where the areas that are largely rural, with widespread subsistence farming and high human–livestock contact, conditions conducive to vector proliferation and transmission. Therefore, these findings emphasize the expanding ecological niche of rickettsioses beyond the traditional endemic zones. The lower recorded incidence in the Western and more urbanized districts is likely multifactorial and should not be attributed solely to reduced rural exposure. Several alternative, non-mutually exclusive explanations warrant consideration. First, a genuinely lower abundance of trombiculid mite vectors and their rodent reservoir communities is expected in built-up, low-vegetation environments. Second, the dominant circulating agent in peri-urban settings may shift toward murine typhus, whose rat-flea vector persists in urban and peri-domestic environments, but which is under-recognized and only rarely laboratory-confirmed in Sri Lanka. Third, healthcare-seeking behaviour and diagnostic pathways differ between urban and rural populations. Fourth, and importantly, the substantial clinical overlap of rickettsioses with dengue, leptospirosis, and other acute febrile illnesses that are highly prevalent in the Western Province may divert clinical suspicion and laboratory testing away from rickettsial aetiologies, resulting in misclassification and underdiagnosis. Taken together, these possibilities indicate that low recorded incidence in urbanized regions may partly reflect differential case ascertainment rather than a genuine absence of transmission risk. However, sporadic cases across these regions indicate that transmission risk persists even outside high-burden areas. The spatial distribution highlights that the rickettsioses in Sri Lanka is not uniformly distributed and concentrated in distinct ecological and epidemiological hotspots. Therefore, these observations highlight the need for region-specific surveillance and control strategies, particularly targeting Jaffna district and the central hill country, while also maintaining vigilance in emerging southern foci.

The temporal analysis revealed a gradual increase in incidence from 2009 to 2019, followed by a decline in subsequent years. Several factors may explain this pattern. The rise prior to 2019 may be due to enhanced clinical recognition and reporting after rickettsioses were declared notifiable diseases, coupled with possible ecological changes favouring vector proliferation. The declining trend in 2019 could be linked to disruptions in routine health service delivery and disease surveillance during the COVID-19 pandemic, which has been shown to affect reporting of other febrile illnesses in Sri Lanka [39]. Seasonal patterns were also evident, with peak incidence observed at the beginning and end of the year. These periods correspond to the northeast and inter-monsoonal rainfall seasons, which create favourable habitats for vector mites and fleas as highlighted by previous studies that rainfall and humidity strongly influence rickettsial transmission dynamics [33,34].

Similar to trends reported in India, where national surveillance has revealed increasing detection over the past two decades [32,40]. The observed decline trend in recent years could also be due to health system disruptions due to COVID-19 pandemic, a phenomenon also reported for rickettsioses and other febrile diseases in Thailand and Vietnam [41]. Some studies conducted in India and Taiwan have indicated that scrub typhus incidence is highest during cooler or transitional months [31,32]. This reinforces the role of rainfall and temperature seasonality in regulating vector abundance and human exposure.

The climate-linked modeling confirmed that rickettsioses incidence is strongly associated with meteorological factors, consistent with our a priori biological hypotheses. As hypothesized, mean temperature was negatively associated with case incidences, while maximum and minimum temperatures showed positive correlations with the case numbers. This pattern is consistent with our hypothesis that trombiculid mite larval activity peaks during cooler transitional seasons: absolute mean temperature is a poor proxy for seasonal transitions, while temperature extremes better capture the periods when vector activity and human exposure risk converge [31–33]. Rainfall also showed a significant positive association with incidence, consistent with the hypothesized role of post-monsoonal rainfall in promoting dense scrub vegetation and moist microhabitats that support mite larval survival and rodent host abundance [25,34]. Geographic predictors remained significantly associated with both incidence and zero-inflation probabilities, consistent with the known spatial ecology of scrub typhus and spotted fever group rickettsioses across Sri Lanka’s diverse ecological zones, and likely capturing unmeasured district-level determinants such as land use, vegetation type, agricultural practices, and vector abundance.

Comparable findings have been reported in Korea, where scrub typhus incidence increased with temperature fluctuations rather than absolute averages [33]. Similarly, a study conducted in China indicated a positive association between rainfall, humidity, and scrub typhus outbreaks, consistent with the findings in the present study that enhances vector habitats with rainfall [25]. Spatial predictors such as latitude and longitude were also observed significant, highlighting the importance of geographic clustering. The spatial heterogeneity has been described in India, Thailand, and China, where rickettsial transmission varies sharply across neighboring districts depending on land cover and socioeconomic conditions [40,42]. The observed decline in variability of incidence with increasing relative humidity and maximum temperature in the current study suggests greater stability of transmission under such conditions. These findings are important for forecasting, as they indicate that climatic fluctuations could be used as early warning signals for outbreaks. The robust fit of the ZAGA GAMLSS model further strengthens confidence in these associations and demonstrates the utility of advanced statistical frameworks for handling zero-inflated epidemiological data.

From a public health perspective, the identification of high-burden districts offers opportunities for targeted interventions, including community awareness, early diagnostic capacity, and vector control measures. Integration of climate information into surveillance and monitoring programmes may enhance predictive modeling and facilitate proactive responses, particularly in hotspot areas such as Jaffna and the central highlands. As demonstrated in Taiwan and China, where temperature-based forecasting has been used to anticipate outbreaks [25,31]. Importantly, like other countries in the south asian regions, Sri Lanka still faces challenges in laboratory confirmation and differential diagnosis where overlapping symptoms with dengue, malaria, and leptospirosis complicate clinical recognition [42]. Therefore, strengthening diagnostic capacity beyond research laboratories remains a pressing need, given the nonspecific clinical presentation of rickettsioses and its overlap with other febrile illnesses such as dengue and leptospirosis [20,43].

This study has several important limitations that must be considered when interpreting the findings. First, and most critically, the national surveillance system of Sri Lanka reports rickettsioses under a single aggregated category of “typhus fever,” without etiological differentiation between scrub typhus *(Orientia tsutsugamushi*, vector: trombiculid mites), murine typhus (*Rickettsia typhi*, vector: rat fleas), and spotted fever group Rickettsia species (vector: ticks). These are biologically and ecologically distinct diseases with different vector habitats, reservoir hosts, and environmental niches. Aggregating them into a single outcome may obscure species-specific environmental associations and should be interpreted with caution. However, published evidence from Sri Lanka indicates that these diseases are partially geographically partitioned scrub typhus predominating in the Northern and North Central provinces, and spotted fever group in the central highlands so the strong spatial effects detected in our model are broadly consistent with this known geographic structure. We urge the national surveillance system to adopt etiologically specific reporting to enable disease-level environmental analyses in the future. The reliance on passive surveillance data may underestimate true incidence due to underreporting, healthcare access barriers, and clinical misdiagnosis; rickettsial diseases share overlapping presentations with dengue, leptospirosis, and malaria. The laboratory confirmation at the population level was not possible, as confirmatory testing (IFA, PCR) is confined to a few research laboratories. The environmental mechanisms proposed here are inferred indirectly from ecological, district- and time-aggregated climatic associations and are not supported by direct entomological or reservoir-host data. No vector abundance or reservoir-host surveillance data are available at the national level; therefore, they are not included in the study. The reported associations should therefore be interpreted as hypothesis-generating rather than mechanistic, and are constrained by the limitations inherent in aggregated data, where population-level associations may not hold at the individual level. Validating the pathways suggested by current spatial and climatic models will require prospective, field-based studies that integrate entomological surveillance and reservoir-host monitoring coupled with etiologically confirmed human case data. Such integrated eco-epidemiological studies are needed to move from the climate–incidence correlations reported here to a mechanistic understanding of rickettsial transmission in Sri Lanka. The factors such as age, gender, or sex could not be included in the analysis because those demographic data are not reported in the Weekly Epidemiological Reports.

## Conclusion

Rickettsioses in Sri Lanka demonstrate marked spatial and temporal heterogeneity, closely linked to climatic variability. The concentration of cases in the Northern Province is consistent with known scrub typhus ecology, and in the central highlands, it is consistent with spotted fever group typhus, coupled with clear seasonal and climate-associated patterns, emphasizing the need for geographically tailored control strategies. This study is constrained by the inability to disaggregate surveillance data by etiological agent, a limitation that future surveillance reforms should urgently address by implementing etiologically specific reporting and expanding laboratory diagnostic capacity at regional hospitals. Incorporating environmental predictors, including temperature, rainfall, and humidity, into routine surveillance systems may strengthen early warning capacity and improve outbreak preparedness. Future research should integrate ecological, entomological, rodent host abundance, and socioeconomic data, alongside species-level disease confirmation, to develop a more comprehensive understanding of disease risk and guide integrated vector management approaches targeting the specific vectors of each rickettsial disease group.
